# Further delineation of KIDAR syndrome: Two new cases with novel variants, functional analysis of the variants and a comprehensive review

**DOI:** 10.1038/s10038-026-01476-9

**Published:** 2026-04-24

**Authors:** Şule Altıner, Ezgi Gökpınar İli, Ahmet Karer Yurtdaş, Okan Kurtçu, Neslihan Doğulu, Ayşe Öktem, Engin Köse, Fatma Tuba Eminoğlu, Behiye Tuğçe Yıldırım, Ayça Dilruba Aslanger, Gözde Yeşil Sayın, Halil Gürhan Karabulut

**Affiliations:** 1 Ankara University, School of Medicine, Department of Medical Genetics, Ankara, Turkey; 2 Başakşehir Çam and Sakura City Hospital, Genetics Diseases Center, İstanbul, Turkey; 3 Ankara University, School of Medicine, Department of Pediatric Metabolism, Ankara, Turkey; 4 Ankara University, School of Medicine, Department of Dermatology, Ankara, Turkey; 5 İstanbul University, İstanbul Faculty of Medicine, Department of Medical Genetics, İstanbul, Turkey

**Keywords:** Genetics, Clinical genetics, RNA splicing

## Abstract

The syndrome known as KIDAR (keratitis, ichthyosis, deafness, autosomal recessive) is extremely rare. It is caused by biallelic mutations in *AP1B1*, encoding adaptor-related protein complex, beta-1 subunit. AP1 complex takes part in the formation of vesicles and the selection of cargo proteins in the trans-golgi network. It also contributes to vesicular transport of ATP7A and ATP7B. Accordingly, KIDAR has been defined as both an adaptinopathy and a copper metabolism disorder. Eleven cases have been reported to date. We report two new KIDAR cases with novel splice site variants—c.1796+1 G > T and c.1796+1 G > C—in *AP1B1* (NM_001127.4) gene. Functional analysis of the first variant revealed that this mutation disrupts the normal splicing process, resulting in the creation of a cryptic donor site 150 base pairs downstream of the canonical donor site which introduces a premature stop codon into the transcript. We also present a review of previously reported KIDAR cases and genetic disorders involving altering copper metabolism and highlight our patients’ new clinical features, which may broaden the recognized phenotype. A detailed study of these cases may contribute to the ongoing genetic and clinical characterization of KIDAR syndrome.

## Introduction

The adaptor protein (AP) complex is composed of heterotetrameric proteins that mediate intracellular membrane trafficking and play a pivotal role in maintaining cellular homeostasis through vesicle formation and cargo selection. The structure of the AP-1 complex highlights the unique and essential role of the β1 subunit (AP1B1). The complex can change its function by using different μ subunits; for example, μ1A1 works in general sorting, while μ1β is responsible for basolateral targeting in epithelial cells. However, β1 remains conserved across all AP-1 complexes and is the only beta-adaptin they use, with its primary role is to recruitment of clathrin. As a result, all AP-1 dependent pathways, including basolateral sorting, rely on β1. This shows that β1 is critical for maintaining proper cell polarity. This feature sets AP-1 apart from other adaptor complexes. AP-2 uses a different beta subunit, β2 for endocytosis at the plasma membrane. AP-3 has tissue-specific β and μ subunits, which provides more functional flexibility [1, 2].

Biallelic pathogenic variants in *AP1B1* cause autosomal recessive keratitis ichthyosis deafness (KIDAR) syndrome (MIM#242150), an extremely rare phenotype characterized by keratitis, ichthyosis, and sensorineural hearing loss. Failure to thrive, global developmental delay, photophobia, low plasma ceruloplasmin and copper levels may accompany keratitis, ichthyosis, and sensorineural hearing loss [3].

We report below two KIDAR cases, with functional analysis of the mutation in the splice region. To date, only eleven cases of this ultra-rare syndrome have been reported. These new cases will contribute to the clinical and genetic characterization of KIDAR syndrome.

## Case reports

### Case 1

The proband was a 13-year-old male, born to consanguineous parents, referred to the outpatient medical genetics department for developmental delay, hearing loss, ichthyosis, and palmoplantar keratoderma. He was born by vaginal delivery at 37 weeks of gestation. His birth weight was 2850 g. His medical history was remarkable for developmental delay, with holding his head unsupported at 1 year, unsupported sitting at 18 months, and independent walking at 3 years. At 1 year of age, he was diagnosed with profound bilateral sensorineural hearing loss and received cochlear implants at 3 years old. He had delayed speech, barely speaking 10 words at age 13. At three months of age, he developed exfoliative erythroderma, slowly progressed over years, and was diagnosed with ichthyosis.

**Fig. 1:**
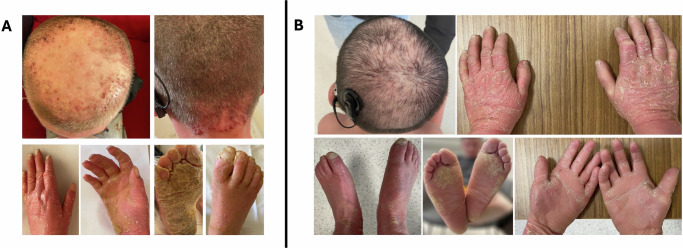
Skin findings of cases. **A**: Skin findings of the case 1 showing scarring alopecia, palmoplantar keratoderma, dysplastic nails, generalized ichthyosis and scaly skin. **B**: Skin findings of the case 2 showing sparse hair with partial alopecia, scaly skin, and palmoplantar keratoderma

Clinical examination revealed an intellectual disability. He had mild facial dysmorphism with a high anterior hairline, sparse eyebrows, microphthalmia, and inward strabismus on the right. He had generalized ichthyosis and palmoplantar keratoderma, scaly skin, and scarring alopecia. He also had hypoplastic teeth and dysplastic nails (Fig. 1A). He had photophobia and corneal scarring. His walk was broad-based, and deep tendon reflexes increased. He had mild mitral regurgitation and patent foramen ovale. Values reported in a complete blood count (CBC), routine biochemical analysis, and very long chain fatty acids were within normal limits.

### Case 2

The patient was a ten-year-old male who was referred to the outpatient clinic for congenital ichthyosis. After an uncomplicated pregnancy, he was born at the 36th week via caesarean section. He was admitted to the newborn intensive care unit for aganglionic megacolon and congenital ichthyosis after birth. Developmental milestones were delayed with independent walking at 3 and speech at 5 years of age. During evaluations for delayed language development at the age of 4, bilateral sensorineural hearing loss was noted. On physical examination, he had a high anterior hairline, sparse eyebrows, sparse hair with partial alopecia, scaly skin, and palmoplantar keratoderma (Fig. 1B). He also had central corneal opacity and photophobia. His CBC and biochemistry values were normal. The patient was born to third-degree consanguineous parents. A brother had died due to aganglionic megacolon shortly after birth. The brother also had congenital ichthyosis.

## Methods

### Ethics approval and informed consent

Written informed consents for genetic investigation and publication were obtained from the parents of the patients. Ethics approval for this study was granted by Ankara University Faculty of Medicine Human Research Ethics Committee (Decision numbers: İ07-508-23, İ11-835-24).

### Sample collection and preparation

Peripheral blood samples were obtained from both patients. Genomic was extraxted using standard laboratory protocols. Total RNA was isolated from peripheral blood of case 1 for transcript analysis. DNA and RNA extraction procedures were performed according to standart laboratory protocols.

### Next-generation sequencing (NGS) and whole-exome sequencing (WES)

For case 1, whole exome sequencing was performed using the QIAseq Targeted DNA Custom Panel kit (Qiagen), and the sequencing was carried out on the Illumina MiSeq® platform with corresponding reagent kits. For case 2, whole-exome sequencing libraries were prepared using the TWIST 36 Mb Human Core Exome Capture Kit following the manufacturer’s recommendations, and sequencing was conducted on the MGI DNBSEQ-G400 system. More than 98% and 97% of targeted exonic regions achieved ≥20x coverage for cases respectively.

Bioinformatic analysis focused on genes associated with congenital ichthyosis. Variant filtering excluded alleles with a minor allele frequency (MAF) ≥ 1% in population databases. Priority was given to rare homozygous variants consistent with the suspected autosomal recessive inheritance pattern. Identified candidate variants were evaluated using standard ACMG pathogenicity criteria and relevant databases.

### Sanger sequencing confirmation and segregation analysis

Candidate variants in *AP1B1* (NM_001127.4) were validated by Sanger sequencing. PCR amplification was performed using *AP1B1*-specific primers (forward: 5’-GGCGGTTGGGAGTAGAGAC-3’; and reverse: 5’-CACCTGAAGAATGGGGACATTT-3’). PCR reactions were prepeared with Amplitaq Gold^TM^360 Master Mix (Thermo Fishes Scientific). Sequencing reactions were carried out using the BigDye^TM^ Terminator v3.1 Cycle Sequencing Kit, and electrophoresis was performed on Applied Biosystems® 3130 and 3500 Genetic Analyzers following the manufacturer’s protocols. Segregation analyses were completed for available family members.

### Transcript analysis (RT-PCR)

For case 1, reverse transcription PCR (RT-PCR) was performed to assess the effect of the *AP1B1* splice-site variant on mRNA processing. As the variant affects the canonical +1 splice donor site of exon 13, primers were design to detect exon 13 skipping and/or intron 13 retentiton. cDNA synthesis and amplification were conducted using the HiScript® II One Step RT-PCR Kit. RT-PCR amplification was performed using AP1B1 primers targeting the exon 12-13 junction (forward: 5’- GGCCACTCAGGACTCAGATA-3’; reverse: 5’-GCTGTGGACCTTCTTGGCGGT -3’). RT-PCR products were purified and sequenced by Sanger sequencing.

## Results

Targeted analysis of rare homozygous variants in the genes associated with congenital ichthyosis revealed homozygous c.1796+1 G > T (case 1) and c.1796+1 G > C (case 2) variants in the *AP1B1* (NM_001127.4) gene.

The variants were not previously reported in the literature or in ClinVar in patients, to our knowledge. c.1796+1 G > A was reported three times as heterozygous state in the gnomAD database, but it has not been reported as homozygous form in population databases (the genome aggregation database (gnomAD), dbSNP, 1000 genomes, NHLBI Exome Sequencing Project (ESP) cohort, and ExAC consortium). c.1796+1 G > C was not reported as heterozygous or homozygous state in population databases.

**Fig. 2:**
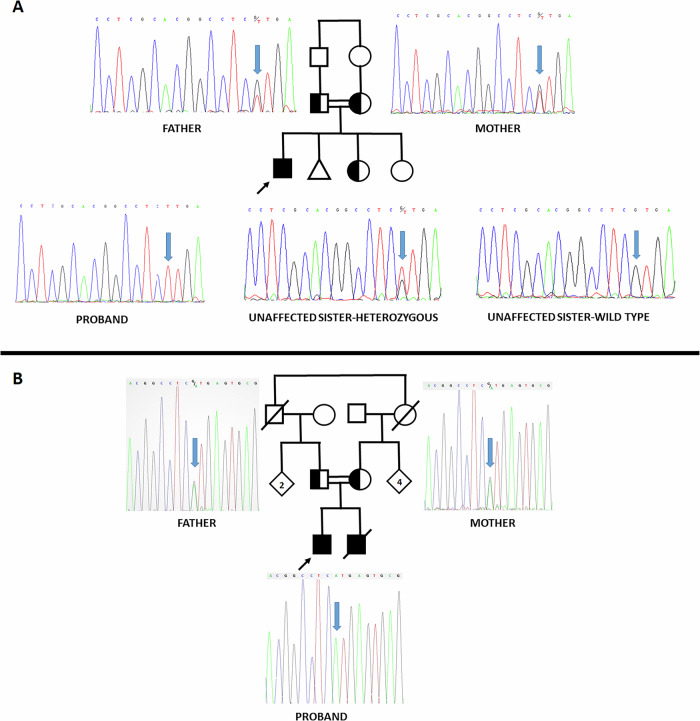
Pedigree of the families and partial electropherograms of the individuals. **A**: The father and mother are heterozygous for the c.1796+1 G > T mutation; the proband is (case 1) homozygous for the mutated allele (arrows). **B**: The father and mother are heterozygous for the c.1796+1 G > A mutation; the proband is (case 2) homozygous for the mutated allele (arrows)

The variants are splice site variants predicted to cause loss of function. Segregation analysis of the variants confirmed autosomal recessive inheritance (Fig. 2A, B).

Briefly, the variants were classified as pathogenic (PVS1, PM2, PP4) according to ACMG 2015 guidelines [4].

**Fig. 3:**
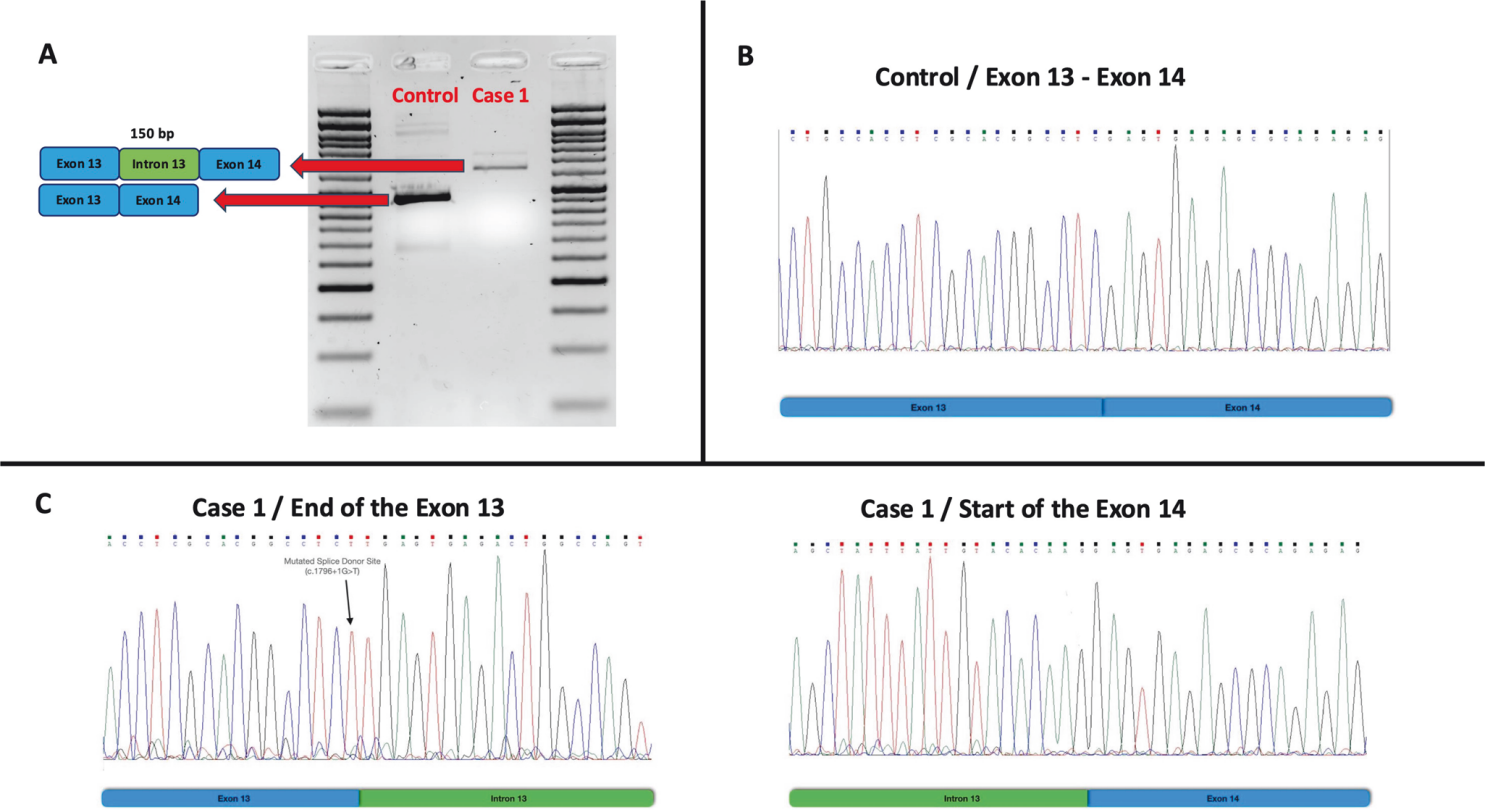
RNA analysis of case. **A**: Gel image, **B**: Electropherogram of the control, **C**: Electropherogram of case 1 showing intronic retention

Sanger sequencing of the RT-PCR product of case 1 revealed retention of the first 150 bp of intronic region in the mature mRNA (Fig. 3).

## Discussion

### Overview and relevance of copper in human physiology

Copper is an essential trace element required for proper functioning of metabolic pathways and for the development and maintenance of central nervous system development. The copper-transporting ATPases ATP7A and ATP7B coordinate copper absorption, cellular uptake, intracellular trafficking, and excretion [5].

Adaptor protein complexes, including AP-1, involved in vesicular trafficking and are required for correct intracellular localization of ATP7A and ATP7B. Disruption of AP-1 function therefore affects both vesicular trafficking and copper metabolism, and this mechanistic link underlies the clinical features shared among AP-1–related disorders [6].

### Genetic disorders related to copper metabolism

The spectrum of copper metabolism disorders includes a range of clinical entities, primarily affecting hepatic and neurologic systems due to disturbed copper levels. Menkes disease (MIM #309400) and Wilson disease (MIM #277900) represent the best-established copper related conditions. Menkes disease, caused by *ATP7A* mutations, is an X-linked disorder leading to severe copper deficiency and progressive neurodegeneration. A milder allelic condition, Occipital Horn Syndrome (MIM #304150), and an ATP7A-related distal motor neuropathy (MIM #300489) have also been described [7]. Wilson disease conversely, is an autosomal recessive disorder caused by *ATP7B* mutations, resulting in copper accumulation in the liver and brain manifesting with hepatic and neurologic findings [8].

Among copper-metabolism disorders beyond ATP7A- and ATP7B-related diseases, the AP-complex–associated conditions MEDNIK and KIDAR syndromes are particularly notable, MEDNIK syndrome (MIM #609313), caused by biallelic pathogenic variants in *AP1S1*, exemplifies a prototypical adaptinopathy, presenting with combined clinical and biochemical features reminiscent of both Menkes and Wilson diseases [9]. KIDAR Syndrome (MIM # 242150), caused by biallelic *AP1B1* mutations, has basic commonalities with MEDNIK [3]. Although limited in number, reported patients with KIDAR syndrome exhibit features including ichthyosis, developmental delay, sensorineural hearing loss, and alterations in copper and ceruloplasmin levels (Table 1).

**Table 1:** Presentation of eleven previous patients with KIDAR and the two patients of this study

	Case 1*	Case 2*	Alsaif [3]	Alsaif [3]	Alsaif [3]	Boyden [10]	Boyden [10]	Vornweg [11]	Ito [12]	Meriç [13]	Faghihi [14]	Vasconcelos [15]	Kaniganti [16]	All patients (N: Feature detected/total)
**Sex (Female:Male)**	Male	Male	Female	Male	Male	Male	Female	Female	Male	Female	Male	Male	Male	4:9
**Age**	13 years	10 years	4 years	1 year	4 years	33 years	New born	2 weeks	2 years	11 months	6 years	14 years	8 weeks	between newborn and 33 years
**Photophobia**	+	+	N/A	N/A	N/A	+	+	+	N/A	+	+	+	N/A	8/8
**Corneal scarring**	+	+	−	N/A	N/A	+	N/A	−	N/A	−	+	+	N/A	5/8
**Ichthyosis, generalized**	+	+	+	+	+	+	+	+	+	+	+	+	+	13/13
**Erythroderma**	+	+	+	+	+	+	+	+	+	+	+	+	+	13/13
**Palmoplantar keratoderma**	+	+	−	−	+	+	+	+	+	+	+	+	−	10/13
**Dystrophic nails**	+	+	−	−	−	+	N/A	−	N/A	−	+	N/A	−	4/10
**Sparse hair**	+	+	+	+	+	−	+	−	−	+	+	+	+	10/13
**Sparse eyebrows**	+	+	−	−	+	N/A	+	−	N/A	+	+	+	+	11/11
**Alopecia, partial**	+	+	−	−	−	−	+	+	−	+	−	+	−	6/13
**Scaly skin**	+	+	−	−	+	−	−	−	−	+	+	−	−	5/13
**Other skin/nail/hair problems**	scarring alopecia	−	wiry texture hair	wiry texture hair, hairline recession		anhidrosis	thick yellow scale on the scalp					epiblepharon	generalized wrinkling and desquamation of skin	wiry texture hair (N:2), thick yellow plate−like scale on scalp (N:1), hairline recession (N:1), anhidrosis (N:1), papules (N:1), scarring alopecia (N:1), wrinkling skin (N:1)
**Hepatopathy**	−	−	+	+	−	N/A	N/A	−	−	+	+	+	+	6/11
**Enteropathy**	−	−	+	+	N/A	N/A	N/A	+	+	+	−	+	−	6/10
**Hearing loss (bilateral sensorineural)**	+	+	+	+	+	+	+	+	+	+	+	+	+	13/13
**Dysmorphism**	high anterior hairline, microphthalmia, inward strabismus, hypoplastic teeth	high anterior hairline		frontal bossing, narrow and incomplete cleft of the soft palate	frontal bossing			ectropion				frontal prominance, ectropion, median cleft palate,narrow palpebral fissures, narrow nasal ridge, base and nares, smooth philtrum, down−turned corners of mouth, generalized microdontia, prominent abdomenand umbilicus, diastasis recti, clubbing of hands and feet	arge ear lobes,frontal bossing with a high anterior hairline, underdeveloped supraorbital ridges, maxillary hypoplasia, and a depressed nasal bridge with anteverted nares,hypertelorism	bilateral ectropion (N:3), frontal bossing (N:4), high anterior hairline (N:3), microphthalmia (N:1), inward strabismus (N:1), hypoplastic teeth (N:1), incomplete cleft of the soft palate (N:1), gingival destruction (N:1), fissured tongue (N:1), tooth loss (N:2), smooth philtrum (N:1), depressed nasal bridge with anteverted nares (N:1), down−turned corners of mouth (N:1)
**Global developmental delay**	+	+	+	+	+	+	+	+	+	+	+	+	+	13/13
**Intellectual disability**	+	+	+	N/A	+	−	N/A	N/A	+	+	−	+	N/A	7/9
**Brain MRI**	N/A	N/A	N/A	cerebral atrophy, thin corpus callosum	N/A	N/A	N/A	N/A	N/A	normal	corpus callosum hypoplasia	cerebral atrophy, thin corpus callosum	delayed myelination, mild supratentorialventricular enlargement, and narrowed internal auditorycanals	cerebral atrophy (N:2), corpus callosum hypoplasia (N:2), normal (N:1), delayed myelination (N:1), mild supratentorial ventricular enlargement (N:1)
**Complete blood count**	normal	normal	N/A	anemia	N/A	trombocytopenia	trombocytopenia	normal	N/A	normal	normal	anemia, neutropenia, trombocytopenia	neutropenia, trombocytopenia	trombocytopenia (N:2), anemia (N:1), normal (N:6)
**Biochemical analysis**	normal	normal	elevated transaminase and elevated alkaline phosphotase	elevated transaminase and elevated alkaline phosphotase	normal	N/A	N/A	normal	elevated alkaline phosphotase	normal	elevated transaminase and elevated alkaline phosphotase	elevated transaminase	elevated transaminase, elevated gamma glutamyl transferase	variable elevated alkaline phosphotase (N:5), variable elevated alaline transaminase (N:5), normal (N:4)
**Mildly elevetad VLCFAs levels**	normal	normal	+	+	−	N/A	N/A	−	N/A	−	−	N/A	−	2/7
**Low plasma copper level**	N/A	N/A	+	+	+	N/A	N/A	−	+	−	−	+	−	5/9
**Low plasma ceruloplasmin level**	N/A	N/A	+	+	+	N/A	N/A	−	+	−	−	+	−	5/9
**Other features**	mild mitral regurgitation, patent foramen ovale, broad based gait, increased deep tendon reflexes	aganglionic megacolon	brisk deep tendon reflexes	respiratory infections, hypoplastic scrtoum with undescended testes		meningitis, gingival destruction, fissured tongue, bone marrow biopsy revealed dysmegakaryopoesis	nistagmus		recurrent infection, intussusception	short stature, febrile seizure, high myopia, low T4, high tyhroid-stimulating hormone and reduced response to grwoth hormone stimulation test	respiratory infection, hypotonia	prominent abdomen and umbilicus, diastasis recti, inguinal hernia, bilateral cryptorchidism with testicular atrophy and left hydrocele clubbing of hands and feet, corneal leukoma and astigmatism	growth delay, recurrent infection, seizures, hypotonia, elevated urinary copper, elevated liver copper	

^*^Cases from present literature

*MRI* magnetic resonance imaging, *N*number, *N/A*not available, *VLCFAs*very long chain fatty acids

### Identified genetic variants and their functional impact

This report presents two novel *AP1B1* homozygous variants at the same position (c.1796+1 G > A and c.1796+1 G > T; NM_001127.4) both affecting the donor splice site. Bioinformatic prediction tools GeneSplicer and VarSEAK indicated a deleterious effect. RNA analysis showed mutations abolish the authentic splice donor site and promote activation of a cryptic donor site 150 bp downstream, resulting in partial intronic retention within the mature mRNA.

The retained intronic sequence disrupts the reading frame and introduces an early premature termination codon (TGA), predicted to produce a truncated, non-functional AP1B1 protein or trigger nonsense-mediated decay. These findings provide strong molecular evidence of a loss-of-function mechanism consistent with the established pathophysiology of KIDAR syndrome.

### Genotype–phenotype correlation in KIDAR syndrome

The clinical manifestations observed in our patients align closely with the phenotypic spectrum reported in all previously described KIDAR cases. Both of our patients exhibited the characteristic KIDAR phenotype, including generalized ichthyosis, erythroderma, bilateral sensorineural hearing loss, and global developmental delay, all of which have been consistently reported in previously published cases [3, 10–16] as summarized in Table 1. Furthermore, photophobia and corneal scarring, delineated in detail by Boyden et al. [10] Meriç et al. [13] and Faghihi et al. [14] were also present in our cases.

Dysmorphic features demonstrate notable heterogeneity across published reports, with findings such as high anterior hairline, frontal bossing, and ectropion recurrently described [2, 11, 15, 16]. The mild dysmorphic traits identified in our patients are consistent with this phenotypic variability.

Low serum copper and ceruloplasmin levels, reported in several studies [3, 12, 15, 16], could not be evaluated in our patients because irregular follow-up prevented appropriate laboratory testing. In contrast, mildly elevated VLCFA levels, as described by Alsaif et al. [3] were not detected in our patients. Collectively, these observations confirm that our cases exhibit the core phenotype characteristic of KIDAR syndrome.

### Novel clinical findings and potential phenotypic expansion

In addition to the canonical KIDAR phenotype, our patients displayed atypical and previously unreported manifestations. Case 1 had mild mitral regurgitation, patent foramen ovale, a broad-based gait pattern, and increased deep tendon reflexes; while case 2 had histopathologically confirmed aganglionic megacolon. Case 2 and his deceased sibling had aganglionic megacolon in addition to congenital ichthyosis. Whole exome sequencing did not reveal any findings associated with aganglionic megacolon in case 2. Furthermore, chromosomal microarray analysis, despite being performed under low QC metrics due to poor DNA quality, did not reveal any gross genetic abnormality. It is possible that the siblings have a second genetic anomaly at an as yet unidentified locus associated with aganglionic megacolon. On the other hand, it is noteworthy to report the co-occurance of aganglionic megacolon and KIDAR phenotype in both siblings for enteric nervous system involvement may be an emerging, previously unrecognized component of KIDAR syndrome. Additional studies will be essential to determine whether this association is consistent.

### Evolutionary conservation and potential mutational hotspots

**Fig. 4:**
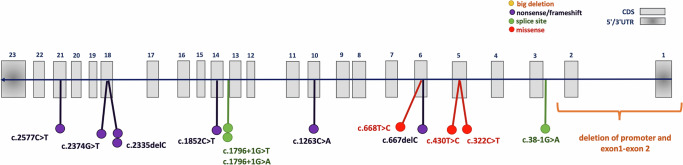
Schematic representation of the *AP1B1* gene (NM_001127.4) and distribution of reported variants. The gene is shown with 23 exons (gray boxes), where light gray indicates CDS regions and shaded boxes indicate 5′ and 3′ UTRs. The transcript orientation is indicated by the arrow. Reported variants are mapped to their respective exons and color-coded by variant type: nonsense/missense variants (purple), splice-site variants (green), missense variants (red), and large deletions (orange). A promoter and exon 1–2 deletion is also indicated. Reported mutations include large deletions affecting the promoter and exon 1–2 [3, 15], splice-site variant c.38-1 G > A [3], nonsense variants including c.2374 G > T (p.Glu792*) [10], c.1852C>T (p.Gln618*) [12], and c.2577 C > T (p.Gln893*) [12], frameshift variants c.2335delC (p.Leu779Serfs*26) [10], c.667delC (p.Leu223Trp*fsTer38) [16], missense variants c.430 T > C (p.Cys144Arg) [10], c.1263 C > A (p.Thr421*) [14], c.668 T > C (p.Leu223Pro) [13], and c.322 C > T (p.Arg108Trp) [11] have been described. Also splice site variants c.1796+1 G > T / c.1796+1 G > A from present study indicated

Multiple alignments of 99 vertebrate genomes demonstrate that the affected splice region is highly conserved (Phylo100way score:7.905), supporting its functional importance. Among the eleven previously reported *AP1B1* variants, only one splice site mutation has been described (Fig. 4).

While no mutational hotspots have been definitively identified in *AP1B1* to date, the presence of two different pathogenic variants at the same splice position in unrelated families raises the possibility that this site may be particularly vulnerable to disruption. Further variant accumulation will be necessary to determine whether this region represents a recurrent hotspot.

## Conclusion

This study reports two previously unreported splice-disrupting *AP1B1* variants and providing transcript-level evidence for aberrant splicing with cryptic donor-site activation. The integration of clinical, molecular, and RNA-level data strengthens the causal inference between AP1B1 loss of function and the multisystem phenotype, while the observation of enteric nervous system involvement offers a biologically plausible extension of the disease mechanism that has not been previously emphasized in the literature.

Despite these advances, certain constraints should be acknowledged. The absence of biochemical copper parameters, due to inconsistency in clinical follow-up, limits direct correlation of the observed genotype with copper metabolism profiles. In addition, although aberrant splicing was demonstrated at the transcript level, the study lacks protein-level validation and downstream assays capable of interrogating AP-1–mediated trafficking defects at a cellular or subcellular resolution. Future research directives should include longitudinal clinical surveillance, systematic phenotyping across organ systems, and functional characterization of AP1B1 deficiency using cellular models or patient-derived tissues. High-resolution imaging of vesicular dynamics, coupled with proteomic and transcriptomic analyses, would be particularly informative for refining mechanistic understanding and establishing more robust genotype–phenotype correlations.

In summary, the identification of two novel pathogenic splice-site variants in *AP1B1* provides compelling evidence for an expanded mutational spectrum in KIDAR syndrome and highlights a potential mechanistic link between AP-1 dysfunction and impaired enteric neural crest development. The delineation of aganglionic megacolon as a possible syndromic component further underscores the systemic impact of AP1B1 deficiency and raises important considerations for clinical evaluation, surveillance, and genetic counseling. Collectively, these findings enhance diagnostic precision, extend the recognized phenotypic boundaries of the disorder, and offer new insights into the molecular pathology underlying AP-1–related adaptinopathies.

## Data Availability

The data that support the findings of this study are available from the corresponding author upon reasonable request.

## References

[1] Bonifacino JS, Traub LM. Signals for sorting of transmembrane proteins to endosomes and lysosomes. Annu Rev Biochem. 2003;72:395–447. 12651740 10.1146/annurev.biochem.72.121801.161800

[2] Fölsch H, Ohno H, Bonifacino JS, Mellman I. A novel clathrin adaptor complex mediates basolateral targeting in polarized epithelial cells. Cell. 1999;99:189–98. 10535737 10.1016/s0092-8674(00)81650-5

[3] Alsaif HS, Al-Owain M, Barrios-Llerena ME, Gosadi G, Binamer Y, Devadason D, et al. Homozygous loss-of-function mutations in AP1B1, encoding beta-1 subunit of adaptor-related protein complex 1, cause MEDNIK-like syndrome. Am J Hum Genet. 2019;105:1016–22. 31630791 10.1016/j.ajhg.2019.09.020 PMC6848991

[4] Richards S, Aziz N, Bale S, Bick D, Das S, Gastier-Foster J, et al. ACMG Laboratory Quality Assurance Committee. Standards and guidelines for the interpretation of sequence variants: a joint consensus recommendation of the American College of Medical Genetics and Genomics and the Association for Molecular Pathology. Genet Med. 2015;17:405–24. 25741868 10.1038/gim.2015.30 PMC4544753

[5] Bandmann O, Weiss KH, Kaler SG. Wilson’s disease and other neurological copper disorders. Lancet Neurol. 2015;14:103–13. 25496901 10.1016/S1474-4422(14)70190-5 PMC4336199

[6] Maung MT, Carlson A, Olea-Flores M, Elkhadragy L, Schachtschneider KM, Navarro-Tito N, et al. The molecular and cellular basis of copper dysregulation and its relationship with human pathologies. FASEB J. 2021;35:e21810. 34390520 10.1096/fj.202100273RR

[7] De Feyter S, Beyens A, Callewaert B. ATP7A-related copper transport disorders: A systematic review and definition of the clinical subtypes. J Inherit Metab Dis. 2023;46:163–73. 36692329 10.1002/jimd.12590

[8] Immergluck J, Grant LM, Anilkumar AC. Wilson Disease. 2025 Apr 3. In: StatPearls [Internet]. Treasure Island (FL): StatPearls Publishing; 2025 Jan–. [cited 01.19.2026]. Available from: https://www.ncbi.nlm.nih.gov/books/NBK441990/28723019

[9] Martinelli D, Dionisi-Vici C. AP1S1 defect causing MEDNIK syndrome. Ann N Y Acad Sci. 2014;1314:55–63. 24754424 10.1111/nyas.12426

[10] Boyden LM, Atzmony L, Hamilton C, Zhou J, Lim YH, Hu R, et al. Recessive mutations in AP1B1 cause ichthyosis, deafness, and photophobia. Am J Hum Genet. 2019;105:1023–9. 31630788 10.1016/j.ajhg.2019.09.021 PMC6849088

[11] Vornweg J, Gläser S, Ahmad-Anwar M, Zimmer AD, Kuhn M, Hörer S, et al. Compound heterozygous AP1B1 mutations in KIDAR. Br J Dermatol. 2021;184:1190–2. 33452671 10.1111/bjd.19815

[12] Ito Y, Takeichi T, Igari S, Mori T, Ono A, Suyama K, et al. MEDNIK-like syndrome due to compound heterozygous mutations in AP1B1. J Eur Acad Dermatol Venereol. 2021;35:e345–7. 33349978 10.1111/jdv.17098

[13] Meriç R, Ercan-Sencicek AG, Alkaya DU, Sahin Y, Sar M, Bilguvar K, et al. A patient with mental retardation, enteropathy, deafness, peripheral neuropathy, ichthyosis, keratodermia syndrome caused by AP1B1 gene variant. Clin Dysmorphol. 2021;30:54–7. 32969855 10.1097/MCD.0000000000000350

[14] Faghihi F, Khamirani HJ, Zoghi S, Kamal N, Yeganes BS, Dianatpour M, et al. Phenotypic spectrum of KIDAR syndrome. Eur J Med Genet. 2022;65:104449. 35144013 10.1016/j.ejmg.2022.104449

[15] Vasconcelos AP, Nogueira A, Matos P, Pinto J, Pinho MJ, Fernandes S, et al. Severe KIDAR syndrome caused by deletion in the AP1B1 gene: report of a teenage patient and systematic review of the literature. Eur J Med Genet. 2023;66:104827. 37657632 10.1016/j.ejmg.2023.104827

[16] Kaniganti T, Gean-Akriv G, Keidar T, Levy-Shraga Y, Debby A, Grinbery I, et al. Characterization of KIDAR syndrome with a novel AP1B1 variant. Mol Genet Metab. 2025;144:109056. 40101690 10.1016/j.ymgme.2025.109056

